# The Ideal Graduate Study Institution.—What Germany Has Done

**Published:** 1909-07-17

**Authors:** 


### Post-Graduate Medical Section.

### THE IDEAL GRADUATE STUDY INSTITUTION.—WHAT GERMANY HAS DONE.

### II.—THE "KAISERIN FRIEDRICH ENDOWMENT."

The rapid development of the work of the Central Committee, the increase of its activity, and concomitantly, the enlargement of the State Collection of Demonstration Specimens, necessitated a better organisation and a larger and more permanent central domicile than the Committee possessed. The Committee was already in receipt of an annual grant from the Kultus ministerium, and it was decided, in consultation with the ministerial representatives, and upon the instigation of Professor Kutner, to establish a bureau and a permanent home for the collection, and in view of the interest which her late Majesty the Dowager Empress Friedrich had shown in the movement for the promotion of graduate study, it was unanimously decided that this building should be called the "Kaiserin Friedrich Haus." Early in March 1903 the president of the Committee, the late Excellenz Professor von Bergmann, was received in audience by his Majesty the Emperor, and presented the plan of the proposed building with a short exposition of what was suggested. His Majesty was graciously pleased to approve of the specifications, and a few days later, in a letter to Professor von Bergmann, formally accepted the patronage of the institution and expressed his gratification at the desire of the Committee that it should be associated with the name of the late Dowager Empress. On March 7, 1903, the Committee met at the Kultus ministerium and discussed the question of ways and means. It was resolved not to formulate a general appeal, as had been proposed at first, in order to obtain funds for the building and endowment of the institute, but to work privately, and, as a primary essential, to form a strong building committee consisting of those present with power to co-opt more members. Professor Dr. Althoff stated that he had already been promised a sum of £10,000 by a friend in aid of the building fund, and, cheered by this encouragement, the Committee set to work actively and at once. There were two examples to encourage it further. Thus, in America, the New York Post-Graduate Medical School owes its existence to private munificence, while in Russia the fine institutes for post-graduate study associated with the name of the Grand Duchess Helena Paulowna, at St. Petersburg and Moscow, had also been founded, and to a large extent endowed, by private benefactors. It is true, these institutions were not planned on quite so large a scale as this proposed Kaiserin Friedrich Haus, which was to serve not only as a monument to the generosity and interest of the late Dowager Empress but which was to be worthy of the large organisation which had been developed during the past few years and worthy the dignity of the medical profession in the German Empire. But what had been done in America and Russia could surely be rivalled in Germany, unless the medical profession and its friends were too lukewarm to make the attempt, and that no member of the Committee was unpatriotic enough to believe. The result has proved that this trust of the Committee in the solidarity and active interest of the medical profession was amply justified. By the end of May 1903—thus barely three months after the initial meeting—more than £50,000 had been subscribed, and with this it was decided to start building operations. Subscriptions came from all quarters, and it is satisfactory to note that those who responded best to the appeal (which, in accordance with the original resolution, was made privately) were the members of the medical profession, though the large manufacturing firms and several wealthy laymen contributed generously, while his Majesty the Emperor showed his active interest in the movement by becoming one of the donors to the fund. With the approval of the Emperor the Committee decided to use the money so collected to found the "Kaiserin Friedrich Endowment for the Promotion of Graduate Study in Medicine," the objects of which were primarily to build a central domicile for the State Collection, which would at the same time serve as a central bureau for the organisation. As formulated in the original "charter" the Endowment is constituted as follows:

"It is under the direct patronage of the Minister for Education, and its management is vested in a curatorium composed of ordinary and honorary members. Each donor to the fund (by donors being meant those who have subscribed 10,000 marks towards the endowment) is an ordinary member, and a list of such donors is inscribed on a marble tablet in the hall of the institute. The other ordinary members of the curatorium consist of the executive of the Central Committee, two representatives of the Minister for Education, and a representative of the University of Berlin. Honorary members are such persons as in the opinion of the curatorium may have worked in the interests of post-graduate study and by their writings or actions contributed to its advance and progress. From the members of the curatorium are chosen, every three years, a chairman, a vice-chairman, a secretary and a treasurer. As at present constituted the chairman is Wirk. Geh. Rat. Dr. Von Bitter, the vice-chairman Geh. Kommerzienrat E. von Mendelssohn-Bartholdy, the secretary Professor Dr. R. Kutner, and the treasurer Consul-General R. von Mendelssohn. At present the only two honorary members are Dr. Studt, ex-Minister for Education. The general meeting of the curatorium takes place annually on the anniversary of the birthday of the late Dowager Empress Friedrich (November 21), when the annual report is presented." With this report we will deal later on.

Having formulated the rules and regulations, the committee, or, as one should now style it, the curatorium, proceeded to carry out the design. An excellent site was fortunately available, namely, a corner block on the Luisen platz, fronting the Platz itself and lying alongside Hannover Street. This, almost an ideal site, as it was central and close to the main hospitals, was acquired at a cost of 689,000 marks, and the building committee accepted the original plans which had been approved of by the Emperor. Building operations were commenced in July 1904, and in March 1906 the Kaiserin Friedrich Haus was formally opened. The total cost, including purchase of the original site, taxes, specifications, furniture, etc., was approximately £55,120. The surplus of the collected funds was used as the nucleus of a permanent endowment. A certain amount had been spared by the munificent donations of instruments, furniture and apparatus presented to the institution by various firms and private benefactors. Before proceeding to the detailed description of this interesting institution, it may be instructive to give a short account of the present financial position of the "Kaiserin Friedrich Haus," and to show how it is kept up. The revenue of the institution is derived from four main sources, irrespective of occasional subscriptions and donations. These main sources are (1) the annual grant made to the institution for the upkeep of the State Collection by the Kultus ministerium; (2) the annual income derived from the rental of exhibition spaces in the permanent exhibition rooms; (3) rental accruing from occupied houses belonging to the institution on Hannover Street, standing on the block purchased and not used for the building, and (4) interest on the nucleus of the permanent endowment fund. From these sources the institution derives an average annual income of 43,000 marks, and as at present the annual expenditure is calculated at 40,000 marks the institution may be regarded as thoroughly successful from a financial point of view.

But financial success is not the only, nor indeed the main point that is necessary to ensure the complete success of such an undertaking. Were the interest of the mass of the profession wanting, the institution could not be said to be a success, and it is satisfactory to note that this interest is maintained and is daily increasing. The house on the Luisen platz is a centre for medical Germany. Once within its doors, the practitioner feels that he is in his own domain. This is his palace; here lie his interests, here is the centre for his graduate study. To a very large extent this success is due to the unfailing courtesy and consideration of the staff. This institution, in fact, is more than German; it is in a sense international. Go up to the bureau, no matter from what part of the world you come so long as you are a medical practitioner, you will be treated as a colleague and a brother, and patiently and impartially you will be informed of any course you may be interested in. The Committee is making strenuous efforts to render its information authentic and complete, and we would earnestly urge post-graduate institutions in England to enter into regular correspondence with the institution, to exchange information with it, and to become acquainted with its work. Only by so doing can we hope to reciprocate the services which the institution has rendered and is daily rendering to English and American graduates in Germany. With the development of the activities of the Committee it became desirable that the institution and especially the Central Committee should have an organ or journal of its own, in order to publish periodical lists of its courses, and so keep practitioners informed of the work that was being done on their behalf. The need for a paper, such as The Hospital, which should appeal in the first place to the busy general practitioner was felt, and in order to supply such an organ, the Committee started its monthly journal, which was soon expanded into a fortnightly. At present this magazine possesses the largest circulation of any German medical paper It is a thoroughly practical paper, appealing to the general practitioner, giving full information regarding the various courses, not only those under the auspices of the Committee, but those organised independently of it, and articles of general interest, abstract of post-graduate lectures, and accounts of foreign graduate courses.

The Committee has throughout received the cordial support of the medical profession, without which it could not have achieved this success. Not less warm and openly expressed has been the support accorded to it by the Emperor and by the State. His Majesty has, on more than one occasion, paid a visit to the Kaiserin Friedrich Haus, inspected the collections, and expressed his gratification at the good work that the Committee is doing, while the State is directly represented on the Curatorium, and by means of its annual contribution has as directly identified itself with the work of the Committee. Their Majesties King Edward and Queen Alexandra, on the occasion of their last visit to Germany, honoured the "Kaiserin Friedrich Haus" by a visit, and expressed to the management of the institution their enthusiastic appreciation of its merits and achievements.

